# Depressive symptoms and suicidality by menopausal stages among middle-aged Korean women

**DOI:** 10.1017/S2045796022000439

**Published:** 2022-08-26

**Authors:** Se Young An, Yejin Kim, Ria Kwon, Ga-young Lim, Hye Rin Choi, Sunju Namgoung, Sang Won Jeon, Yoosoo Chang, Seungho Ryu

**Affiliations:** 1 Center for Cohort Studies, Total Healthcare Center, Kangbuk Samsung Hospital, Sungkyunkwan University School of Medicine, Seoul, Republic of Korea; 2 Institute of Medical Research, Sungkyunkwan University School of Medicine, Suwon, Republic of Korea; 3 Department of The Environmental Health Centre, Wonju Severance Christian Hospital, Yonsei University School of Medicine, Wonju, Republic of Korea; 4 Department of Psychiatry, Kangbuk Samsung Hospital, Sungkyunkwan University School of Medicine, Seoul, Republic of Korea; 5 Department of Occupational and Environmental Medicine, Kangbuk Samsung Hospital, Sungkyunkwan University School of Medicine, Seoul, Republic of Korea; 6 Department of Clinical Research Design & Evaluation, SAIHST, Sungkyunkwan University, Seoul, Republic of Korea

**Keywords:** Depression, menopausal transition, menopause, middle-aged women, suicidality

## Abstract

**Aims:**

There has been increasing evidence of hormonal changes during reproductive events that lead to mood changes. However, studies on the severity of psychological problems according to the menopausal stage are limited. Thus, this study aimed to investigate the association between menopausal stages, depression and suicidality.

**Methods:**

A total of 45 177 women who underwent regular health check-ups between 2015 and 2018 at Kangbuk Samsung Hospital were included. Participants were stratified into four groups (pre-menopause, early transition, late transition and post-menopause) based on the Stages of Reproductive Aging Workshop Criteria. The Center for Epidemiological Studies-Depression scale (CESD) was used to evaluate depressive symptoms, and the degree of depressive symptoms was classified as moderate (CESD score 16–24) or severe (CESD score ⩾ 25). To measure suicide risk, we administered questionnaires related to suicidal ideation.

**Results:**

Overall, the prevalence of CESD scores of 16–24 and ⩾ 25 was 7.6 and 2.8%, respectively. Menopausal stages were positively associated with depressive symptoms in a dose-dependent manner. Multivariable-adjusted prevalence ratios (PRs, 95% confidence intervals) for CESD scores of 16–24 comparing the stages of the early menopausal transition (MT), late MT and post-menopause to pre-menopause was 1.28 (1.16–1.42), 1.21 (1.05–1.38) and 1.58 (1.36–1.84), respectively. The multivariable-adjusted PRs for CESD scores ⩾ 25 comparing the stages of the early MT, late MT and post-menopause to pre-menopause were 1.31 (1.11–1.55), 1.39 (1.12–1.72), 1.86 (1.47–2.37), respectively. In addition, the multivariable-adjusted PRs for suicidal ideation comparing the early MT, late MT and post-menopause stages to the pre-menopause stage were 1.24 (1.12–1.38), 1.07 (0.93–1.24) and 1.46 (1.25–1.70) (*p* for trend <0.001), respectively.

**Conclusions:**

These findings indicate that the prevalence of depressive symptoms and suicidal ideation increases with advancing menopausal stage, even pre-menopause.

## Introduction

Depression affects more than 300 million people worldwide, with a global prevalence of approximately 4.4% (Friedrich, 2017). Women are twice as likely to be diagnosed with depression as men (Albert, 2015), and depressive symptoms are associated with reproductive events such as menstruation, childbirth and menopause (Soares and Zitek, 2008; Smith *et al*., 2015; Hofmeister and Bodden, 2016). A similar trend exists with suicide rates for women during climacteric periods (Kulkarni, 2018).

Prior studies have investigated the prevalence and risk of depression during perimenopause. However, most studies used a binary category of menopause (postmenopausal *v*. premenopausal) and reported a lack of association, possibly due to the low prevalence of depressive symptoms and the use of relatively small sample sizes (Maki *et al*., 2018). The menopausal transition (MT) is a complex process comprising multiple stages, ranging from pre-menopause, early MT, late MT, to post-menopause, all of which accompany changes in female sex hormones and various aspects of physical health (Derby *et al*., 2009; Harlow *et al*., 2012; Kozakowski *et al*., 2017). It is important to identify the critical period within the MT during which women become vulnerable to mental disorders to provide insights into the optimal timing to ensure timely intervention; however, there is still limited evidence on the association between specific menopausal stages and depressive symptoms.

This study aimed to examine the association between menopausal stages and the prevalence of depressive symptoms and suicidality in a large sample of middle-aged Korean women.

## Methods

### Setting and studying the population

The Kangbuk Samsung Health Study was a cohort study of Korean men and women who underwent comprehensive annual or biennial examinations at the Kangbuk Samsung Hospital Total Healthcare Centers in Seoul and Suwon, South Korea (Chang *et al*., 2016).

The study population consisted of women aged 40–65 years who underwent comprehensive health examinations at the Kangbuk Samsung Hospital Total Healthcare Centers in Seoul and Suwon, South Korea, between 2015 and 2018 (*N* = 59 940). Among the 59 940 women, 14 763 were excluded for the following reasons: history of cancer (*n* = 3962); history of hysterectomy or ovariectomy (*n* = 4422); history of premature menopause (*n* = 256); history of artificially-induced menopause due to radiation (*n* = 209), chemotherapy (*n* = 383) or other (*n* = 629); currently receiving hormone replacement therapy (*n* = 1672, as such interventions can affect menopausal symptoms); currently taking an oral contraceptive (*n* = 320) or currently pregnant (*n* = 6); history of mental illness diagnoses such as panic disorder, claustrophobia, depression and others (*n* = 2357); currently taking anxiety drugs (*n* = 703), antidepressants or other neuropsychological medications (*n* = 869); and missing data related to depressive symptoms and suicidality (*n* = 3779). As some individuals met more than one exclusion criteria, the total number of women ultimately included in the study was 45 177.

This study was approved by the Institutional Review Board of Kangbuk Samsung Hospital (IRB No. KBSMC 2020-08-042). The requirement for informed consent was waived because we used non-identified retrospective data that were routinely collected during the health screening process.

### Measurements

Data on demographic factors, socioeconomic status, health behaviours, dietary intake, medical history (including a history of physician-diagnosed mental illness such as panic disorder, claustrophobia or depression) and medication use (including neuropsychiatric medications such as anxiety drugs and antidepressants) were collected using standardised, structured, self-administered questionnaires. Physical activity was assessed using the validated Korean version of the International Physical Activity Questionnaire Short Form (Oh *et al.*, 2007; Ryu *et al*., 2015). Health-enhancing physical activity (HEPA) was defined as physical activity that meets either of the following two criteria: (i) vigorous-intensity activity on 3 or more days per week for a total of 1500 metabolic equivalent (MET) min/week, or (ii) 7 days of any combination of walking, moderate-intensity or vigorous-intensity activities achieving at least 3000 MET min/week.

Sleep duration and quality were assessed using the Korean version of the Pittsburgh Sleep Quality Index (PSQI). Regarding subjective sleep quality, the response categories of the PSQI were very good, fairly good, fairly bad and very bad. The last two categories were defined as poor subjective sleep quality.

Anthropometric parameters and sitting blood pressure (BP) were measured by trained nurses. Obesity was defined as a body mass index (BMI) of ⩾25 kg/m^2^ according to the criteria for obesity in Asians. Hypertension was defined as BP ⩾ 140/90 mmHg or current use of antihypertensive medication.

Fasting blood measurements included glucose, haemoglobin A1c, insulin, high-sensitivity C-reactive protein and lipid profiles. The homeostatic model assessment-insulin resistance (HOMA-IR) was calculated as follows: fasting blood insulin (uU/ml) × fasting blood glucose (mg/dl)/405. Type 2 diabetes mellitus was defined as fasting serum glucose ⩾ 126 mg/dl, haemoglobin A1c ⩾ 6.5% or current use of insulin or medications for diabetes.

### Assessment of menopausal stage

The participants were classified into four groups according to menopausal stage based on the Stage of Reproductive Aging Workshop + 10 criteria as follows: (1) premenopause (having regular menstrual periods), (2) early MT (having a persistent difference of 7 or more days in the length of consecutive cycles), (3) late MT (occurrence of amenorrhoea of ⩾60 days) and (4) post-menopause (amenorrhoea for ⩾1 year).

### Assessment of depression, suicidal ideation and suicidal behaviour

The Center for Epidemiological Studies-Depression (CESD) was used to evaluate depressive symptoms within the past week using the Korean version of the CESD scale; the internal consistency of the Korean version has been reported to range from 0.84 to 0.91. The CESD is composed of 20 items, each rated on a scale from 0 to 3, with total scores ranging from 0 (lowest) to 60 (highest) (Farmer *et al*., 1988; Cho and Kim, 1998; Kim *et al*., 2007). In a previous study validating the Korean version of the CESD scale, the optimal cut-off point was suggested as 24/25, the point that best corresponds to a clinical diagnosis of depression. In contrast, a CESD score of 16 has been traditionally used as an optimal cut-off for the detection of depressive symptoms. Therefore, the presence of depressive symptoms was defined as a CESD score of ⩾16, and the presence of case-level depression was defined by a cut-off score of ⩾25.

Suicidal ideation was also assessed via a self-administered questionnaire with specific questions, such as ‘In the last year, have you ever thought about wanting to die?’ Suicidal behaviour was determined based on a question, ‘Have you attempted suicide in the last year?’. Responses to the questions were dichotomous as either ‘Yes’ or ‘No’.

### Statistical analyses

The characteristics of the study participants were explored according to CESD score categories. CESD scores were categorised as moderately (16–24 points) and severely depressed (⩾25 points). To test for linear trends, the number of categories was used as continuous variables in the regression models.

We estimated the prevalence ratios (PRs) and 95% confidence intervals (CIs) for CESD scores of 16–24 and ⩾25 for the early MT, late MT and post-menopause stages compared to the pre-menopause stage using participants with a CESD score <16 as the reference group in multinomial logistic regression models. Moreover, to evaluate the association of suicidal ideation across menopausal stages, we used a logistic regression model to estimate the odds ratios with 95% CIs for suicidal ideation. We used three models to progressively reduce confounding associations. We initially adjusted for age and then further adjusted for the centre, year of screening examination, smoking status, alcohol intake, physical activity level, total energy intake, BMI, educational level, antihypertensive medication, parity and age at menarche. In addition, to evaluate the effects of psychological symptoms around the period of the MT, sleep duration and sleep quality were adjusted.

Additionally, we performed stratified analyses in pre-specified subgroups defined by BMI (<25 *v*. ⩾25 kg/m^2^), age at menarche (<12 *v*. ⩾12 years), parity (0 *v*. ⩾1 time), sleep quality (good *v*. poor), smoking status (never smoker *v*. ex-smoker or current smoker), alcohol intake (<10 v. ⩾10 g of alcohol per day) and physical activity (no HEPA *v*. HEPA). Interactions by subgroup were tested using likelihood ratio tests comparing models with and without multiplicative interaction terms. All *p* values were two-tailed, and statistical significance was set at *p* < 0.05. We used STATA version 13.0 (Stata Corp., College Station, TX, USA) for the data analyses.

## Results

The mean (standard deviation) age of the 45 177 study participants was 47.3 (6.5) years (online Supplementary Table S1). The prevalence of pre-menopause, early MT, late MT and post-menopause was 49.7, 15.5, 8.7 and 26.1%, respectively. Compared to premenopausal women, participants in the later menopausal stages were more likely to be older and engage in HEPA. They also had higher rates of hypertension and diabetes, BMI, BP, glucose level and HOMA-IR, and were more likely to take dyslipidaemia medication and have unfavourable lipid profiles.

Overall, the prevalence of CESD scores of 16–24 and ⩾25 was 7.6% and 2.8%, respectively (Table 1). Participants with higher CESD scores were more likely to be older, and current smokers, to drink alcohol and have shorter sleep duration and have poor sleep quality. They were also more likely to have higher parity and prevalence of hypertension, diabetes and dyslipidaemia medication use, as well as higher BMIs, glucose levels and HOMA-IR.

**Table 1.** Baseline characteristics of study participants by depression category

Characteristics	Overall	CESD score			*p* for trend
		<16	16–24	⩾25	
Number of participants	45 177	40 486	3419	1272	
Age (years)^a^	47.3 (6.5)	47.2 (6.4)	48.2 (6.9)	48.4 (7.0)	<0.001
Early menarche (%)^b^	3.0	3.0	2.9	2.4	0.319
Parity (%)^c^	7.3	7.2	8.6	9.2	<0.001
Current smoker (%)	1.8	1.6	3.3	3.6	<0.001
Alcohol intake (%)^d^	10.8	10.4	14.8	15.3	<0.001
HEPA (%)	15.2	15.3	14.4	11.8	<0.001
Education level (%)^e^	70.3	71.0	65.0	63.8	<0.001
Diabetes (%)	4.1	3.9	5.3	6.6	<0.001
Hypertension (%)	9.6	9.4	10.9	11.1	0.002
Medication for dyslipidaemia (%)	4.7	4.5	6.2	6.1	<0.001
Poor sleep quality (%)	19.2	16.1	42.1	57.5	<0.001
Sleep duration (h/day)	6.56 (1.13)	6.60 (1.10)	6.27 (1.25)	6.03 (1.38)	<0.001
Body mass index (kg/m^2^)^a^	22.6 (3.1)	22.5 (3.09)	22.7 (3.29)	22.7 (3.45)	0.001
Systolic BP (mmHg)^a^	106.7 (12.7)	106.7 (12.7)	106.8 (12.6)	107.0 (12.7)	0.272
Diastolic BP (mmHg)^a^	67.8 (9.2)	67.8 (9.2)	67.9 (9.0)	68.4 (9.2)	0.027
Glucose (mg/dl)^a^	94.5 (14.2)	94.4 (14.1)	94.9 (13.7)	96.0 (16.4)	<0.001
Total cholesterol (mg/dl)^a^	195.7 (34.0)	195.6 (33.9)	196.1 (33.8)	196.2 (36.1)	0.365
LDL-C (mg/dl)^a^	124.9 (32.8)	124.9 (32.7)	125.7 (32.3)	125.6 (35.6)	0.125
HDL-C (mg/dl)^a^	66.3 (16.3)	66.3 (16.2)	65.7 (16.4)	66.1 (17.0)	0.097
Triglycerides (mg/dl)^f^	79 (59–109)	78 (59–108)	81 (61–111)	80 (59–114)	<0.001
hsCRP (mg/l)^f^	0.04 (0.02–0.07)	0.04 (0.02–0.07)	0.04 (0.02–0.08)	0.04 (0.02–0.08)	0.004
HOMA-IR^f^	1.22 (0.81–1.82)	1.21 (0.81–1.81)	1.25 (0.82–1.88)	1.25 (0.82–1.94)	0.001
Total energy intake (kcal/d)^f,g^	1119.1 (804.6–1481.3)	1118.5 (804.8–1479.5)	1110.4 (789–1497)	1167.9 (833.5–1528.9)	0.051

BP, blood pressure; CESD, Center for Epidemiological Studies-Depression; HDL-C, high-density lipoprotein cholesterol; HEPA, health-enhancing physical activity; HOMA-IR, homeostasis model assessment of insulin resistance; hsCRP, high-sensitivity C-reactive protein; LDL-C, low-density lipoprotein cholesterol.

a Means (standard deviations).

b <12 years.

c ⩾3 times.

d ⩾10 g of ethanol per day.

e ⩾college graduate; data are expressed as.

f Medians (interquartile ranges), or percentages.

g Among 24 840 participants with plausible estimated energy intake levels (within three standard deviations from the log-transformed mean energy intake).

Menopausal stages were positively associated with depressive symptoms in a dose-dependent manner (Table 2). After adjustment for age, centre, year of a screening exam, smoking status, alcohol intake, physical activity level, total energy intake, BMI, educational level, antihypertensive medication, parity and age at menarche, the PR (95% CI) for CESD scores of 16–24 comparing early MT, late MT and post-menopause stages to the pre-menopausal stage was 1.40 (1.26–1.55), 1.32 (1.15–1.51) and 1.78 (1.54–2.06), respectively. The corresponding PR (95% CIs) for CESD scores ⩾25 was 1.50 (1.27–1.76), 1.59 (1.29–1.95) and 2.21 (1.76–2.79), respectively (Table 2, model 1). After further adjustments for sleep quality and sleep duration, and the history of suicidal behaviour, the association between the menopausal stage and depressive symptoms was slightly attenuated although remained statistically significant (Table 2, model 2; online Supplementary Table S2).

**Table 2.** Prevalence ratios^a^ (95% CI) of depression by menopausal stage

	Menopausal stages				*p* for trend
	Pre-menopause	Early transition	Late transition	Post-menopause	
Number	22 454	7011	3924	11 788	
CESD score 16–24					
Cases (%)	1376 (6.1)	582 (8.3)	302 (7.7)	1159 (9.8)	
Age-adjusted PR	1.00 (reference)	1.41 (1.27–1.56)	1.32 (1.15–1.50)	1.82 (1.57–2.10)	<0.001
Multivariate-adjusted PR^a^					
Model 1	1.00 (reference)	1.40 (1.26–1.55)	1.32 (1.15–1.51)	1.78 (1.54–2.06)	<0.001
Model 2	1.00 (reference)	1.28 (1.16–1.42)	1.21 (1.05–1.38)	1.58 (1.36–1.84)	<0.001
CESD score ⩾25					
Cases (%)	479 (2.1)	216 (3.1)	123 (3.1)	454 (3.9)	
Age-adjusted PR	1.00 (reference)	1.51 (1.28–1.78)	1.58 (1.29–1.95)	2.26 (1.81–2.84)	<0.001
Multivariate-adjusted PR^a^					
Model 1	1.00 (reference)	1.50 (1.27–1.76)	1.59 (1.29–1.95)	2.21 (1.76–2.79)	<0.001
Model 2	1.00 (reference)	1.31 (1.11–1.55)	1.39 (1.12–1.72)	1.86 (1.47–2.37)	<0.001

CESD, Center for Epidemiological Studies-Depression; CI, confidence interval; PR, prevalence ratio.

a Estimated from multinomial logistic regression models using CESD scores as outcomes categorised as <16, 16–24 and ⩾25. Multivariable model 1 was adjusted for age, centre, year of screening examination, smoking status, alcohol intake, physical activity level, total energy intake, body mass index, educational level, antihypertensive medication, parity and age at menarche; model 2: model 1 plus an adjustment for sleep duration and quality.

A total of 3236 women (7.16%) reported suicidal ideation. Menopausal stages were positively associated with suicidal ideation (Table 3). There were 1286 (5.73%) cases of suicidal ideation during the pre-menopause stage, 531 (7.57%) during the early MT, 261 (6.65%) during the late MT and 1158 (9.8%) during the post-menopause stage. The multivariable-adjusted PRs for suicidal ideation comparing early MT, late MT and post-menopause stages to the pre-menopause stage were 1.33 (1.20–1.48), 1.15 (1.00–1.33) and 1.61 (1.38–1.87) (*p* for trend <0.001), respectively. Although attenuated after adjusting for sleep duration and sleep quality, the association with the early MT and post-menopause stages remained statistically significant. However, a significantly higher prevalence of suicidal ideation during late MT compared with pre-menopause was no longer observed. After further adjustment for the history of suicidal behaviour, the associations remained virtually unchanged (online Supplementary Table S3). Depressive symptoms (CESD scores ⩾25) showed a tendency towards a consistently higher prevalence of suicidal behaviour across menopausal stages (online Supplementary Table S4). In additional analyses without excluding women with a history of psychiatric diagnosis, the results were slightly attenuated but overall patterns remained similar to those of the original analyses (online Supplementary Table S5). Analyses using binary category (pre- *v*. postmenopausal stages) showed similar patterns (online Supplementary Tables S6 and S7). When we evaluated the prevalence of (i) isolated depression within the past week, (ii) isolated suicidal ideation within the past 12 months and (iii) concurrent depression and suicidal ideation (i.e. women who reported having both depression and suicidal ideation at a given visit) across menopausal stages, overall patterns of association were similar to the findings of the original analyses for all three dependent variables, with the post-menopause stage being associated with the highest prevalence of either isolated or concurrent depression and suicidal ideation (online Supplementary Table S8).

**Table 3.** Odd ratios^a^ (95% CI) of suicidal ideation by menopausal stage

	Menopausal stages				*p* for trend
	Pre-menopause	Early transition	Late transition	Post-menopause	
Number	22 454	7011	3924	11 788	
Cases (%)	1286 (5.73)	531 (7.57)	261 (6.65)	1158 (9.82)	
Age-adjusted OR	1.00 (reference)	1.34 (1.21–1.49)	1.15 (1.00–1.32)	1.66 (1.43–1.93)	<0.001
Multivariate-adjusted OR^a^					
Model 1	1.00 (reference)	1.33 (1.20–1.48)	1.15 (1.00–1.33)	1.61 (1.38–1.87)	<0.001
Model 2	1.00 (reference)	1.24 (1.12–1.38)	1.07 (0.93–1.24)	1.46 (1.25–1.70)	<0.001

CI, confidence interval; OR, odds ratio.

a Estimated from binomial logistic regression models. Multivariable model 1 was adjusted for age, centre, year of a screening exam, smoking status, alcohol intake, physical activity level, total energy intake, body mass index, educational level, antihypertensive medication, parity and age at menarche; model 2: model 1 plus an adjustment for sleep duration and quality.

In the subgroup analyses, the association between the menopausal stage and depressive symptoms differed by obesity, defined as BMI ⩾ 25 kg/m^2^, with a stronger association in obese women than in non-obese women (*p* for interaction 0.002) (online Supplementary Table S9**)**. The association between the menopausal stage and suicidal ideation was stronger in women with alcohol intake ⩾10 *v*. <10 g/day (*p* for interaction 0.014) (online Supplementary Table S10). Otherwise, the associations did not significantly differ among the subgroups.

## Discussion

In this large study of middle-aged Korean women, depressive symptoms and suicidality were significantly associated with menopausal stages and started to increase from the early transition stage. This association remained significant after adjusting for possible confounders. Our results provide the time window during which both depressive symptoms and suicidal ideation are more likely to occur among women in the early to late transition stage who may benefit from appropriate screening measures.

Previous epidemiologic studies have demonstrated that the prevalence and risk of depressive symptoms markedly rise during peri- and post-menopause (Cohen *et al*., 2006; Freeman *et al*., 2006, 2014; Woods *et al*., 2008; Timur and Sahin, 2010; Bromberger *et al*., 2011; Colvin *et al*., 2017). A cross-sectional study of 685 women found that, compared to premenopausal women, perimenopausal and postmenopausal women had a twofold higher risk of developing depressive symptoms (Timur and Sahin, 2010). Another cross-sectional study reported that major depression was significantly higher when women were in the late perimenopausal or postmenopausal period relative to when they were premenopausal or early perimenopausal (Colvin *et al*., 2017). A longitudinal study from the Study of Women's Health Across the Nation (SWAN) cohort of 221 women demonstrated that women were about two to four times more likely to experience a major depressive episode during or immediately after MT, with the highest risk in postmenopausal women (Bromberger and Kravitz, 2011). However, as these previous studies did not differentiate between early and late MT or between perimenopausal and postmenopausal stages, it is unclear whether there are differences in the prevalence of depressive symptoms across menopausal stages. Studies that did incorporate more specific menopausal stages reported that the likelihood of depressive symptoms tended to peak in the late MT, or immediately before menopause, and slowly wanes thereafter, independent of age (Bromberger *et al*., 2007; Woods *et al*., 2008). In a 14-year longitudinal study from the Penn Ovarian Aging Study, the risk of depressive symptoms was higher before the final menstrual period and decreased thereafter (Freeman *et al*., 2014). Our findings are largely consistent with prior findings in that the likelihood of experiencing depressive symptoms markedly increases during the MT, especially towards the later stages compared with the premenopausal period. In addition, the associations with advancing menopausal stages do not seem to be fully explained by increasing age. However, contrary to previous reports, our findings show that the prevalence of depressive symptoms was higher in postmenopausal women than in women in MT. Depression is a multifactorial condition that can be attributed to genetic, psychosocial, demographic or cultural factors (Bailey *et al*., 2019). It is thus possible that some unmeasured factors inherent to our study population that are distinct from those of Western populations, which have been extensively studied previously, may have led to the differences in patterns. Our findings need to be further evaluated in future longitudinal studies of other Asian populations.

Similarly, we found the highest prevalence of suicidal ideation in postmenopausal women among all menopausal stages. There is relatively limited evidence on suicidality in peri- and post-menopausal women; however, our study findings are consistent with several prior studies (Pinto-Meza *et al*., 2006; Kornstein *et al*., 2010). The reasons underlying the heightened risk of suicidal ideation associated with the postmenopausal stage in our study are not clear. A previous study examined the association between suicide attempts and menstrual stage and reported that women who had experienced menopause and amenorrhoea had low oestradiol and progesterone levels, and women with low gonadal hormones had suicide attempts of greater severity (Baca-Garcia *et al*., 2010). Existing evidence supports the association of oestrogen and increased serotonin activity and low serotonergic function due to a drop in oestrogen levels (McQueen *et al*., 1997; Carretti *et al*., 2005; Berman *et al*., 2006), which may increase suicide risk in those with predisposing factors (Arango *et al*., 2001; Oquendo and Mann, 2001), suggesting that suicidality in postmenopausal women may at least partly have neurobiological underpinnings. Environmental factors also play a crucial role in the association between menopause and suicide. In particular, the role of family was found to be significant in women experiencing menopause. The emotional support of a spouse during the climacteric period was an important protective factor, whereas the lack of family support could lead to increased suicide (Murphy *et al*., 2013). Our study is supported by previous studies that found that MT and post-menopause are periods of not only physical but also psychological vulnerability, demonstrating that women in these stages have an elevated likelihood of depression and suicidal ideation. Optimal intervention strategies need to be developed to prevent depression and suicide attempts in this at-risk population.

In our subgroup analysis, we observed a stronger association between the menopausal stage and depressive symptoms in women with a high BMI. Middle-aged women in MT are predisposed to being obese due to the ageing process, changes in hormonal status and/or mood changes that may prompt maladaptive coping behaviours such as stress eating, and obesity may also exacerbate depressive symptoms (Freeman *et al*., 2006; Blumel *et al*., 2015; Schreiber and Dautovich, 2017). Furthermore, obesity is related to increased severity and frequency of menopausal symptoms or physical limitations that may cause considerable discomfort or irritability and, in turn, may negatively affect the psychological well-being of women. A study found that obese women experienced more frequent vasomotor symptoms (hot flushes, sweating and weight gain) than non-obese women, and the association between obesity and vasomotor symptoms was strongest in postmenopausal women (Koo *et al*., 2017). Compared to women with a normal BMI, overweight and obese women were more likely to have physical limitations, which is also significantly associated with depression (Tseng *et al*., 2012). In addition, obesity may be associated with lower self-esteem, although this association may be population-specific. For instance, an inverse association between obesity and depressive symptoms was found in postmenopausal women with lower education levels, whereas no such association was observed in better-educated women (Jasienska *et al*., 2005). Moreover, there are reports that body image, rather than obesity, is associated with lower self-esteem, which can be a significant predictor of depressive symptoms (Kékes Szabó, 2015; Noh *et al*., 2018). A study conducted on midlife women in the USA suggested that depressive symptoms were not directly correlated with weight *per se* in postmenopausal women after adjustment for available confounders (Schreiber and Dautovich, 2017). In Korean culture, which has relatively high societal pressure for thinness, women tend to overestimate their weight status and develop a distorted body image, which is also observed in other East Asian cultures (Noh *et al*., 2018). Therefore, it is likely that being overweight or obese may be much more of a distressing factor for women in Korea compared to those in Western countries. However, given that our study is cross-sectional, the directionality of the association is unclear; further studies should investigate how obesity mediates the association between menopausal stages and depressive symptoms, and whether there are cultural variations in the relationship.

We also found that higher alcohol intake was significantly associated with a higher prevalence of suicidal ideation in the late MT and post-menopause stage. It is uncertain whether alcohol consumption is driven by existing depressive symptoms, or whether it causes or worsens depressive symptoms and leads to suicidal ideation. It has been reported that depression and alcoholism have strong reciprocal relationships and frequently co-occur (Sullivan *et al*., 2005). There are also reports that alcohol consumption may have a causal effect on the development of major depression (Flensborg-Madsen *et al*., 2009; Boden and Fergusson, 2011). While there is a lack of data on the specific effect of alcohol consumption on the association between menopausal stages and depression, there is a plethora of evidence suggesting that drinking is strongly associated with suicidal ideation (Norstrom and Rossow, 2016), and the association is stronger in women than in men (Yi *et al*., 2016). Studies have suggested that as women reach menopause, various environmental or psychosocial factors such as retirement and loss of loved ones, along with changes in body composition, may affect or exacerbate their drinking habits (Milic *et al*., 2018). Based on the data from the SWAN study, although excessive alcohol consumption may not increase during MT, it is a period of instability that may be associated with changes in alcohol-related behaviours (Peltier *et al*., 2020), which may also be driven by affective factors such as negative mood, retained stress and depressive symptoms related to menopause which may trigger suicidal ideation. Nevertheless, due to the cross-sectional nature of our study, it is difficult to draw conclusions regarding the role of alcohol use in the relationship between MT and depressive symptoms. Further longitudinal studies are needed to confirm our findings.

The present study has several limitations. First, due to the cross-sectional design of our study, causality in the association between menopausal stages and depressive symptoms could not be determined. Second, the diagnosis of depressive symptoms was not made clinically by a physician and was based on a self-administered questionnaire. However, the CESD is one of the most widely used tools in population-based research settings to assess depressive symptoms and has good validity and reliability (Luckett *et al*., 2010). Third, we did not consider the effect of vasomotor symptoms due to the lack of data. Previous reports have documented the close associations between depression, sleep and vasomotor symptoms in peri-menopausal women (Eichling and Sahni, 2005; Cohen *et al*., 2006; Alvaro *et al*., 2013; Worsley *et al*., 2014). Future studies should examine whether the observed association between MT and depression is modified by the presence of vasomotor symptoms. Fourth, although past-year suicidal ideation has been widely implemented in psychiatric surveys and epidemiologic studies (Klimes-Dougan *et al*., 2007; Voss *et al*., 2019), it does not allow for estimating the precise onset time of suicidal ideation. In analyses using isolated depression, isolated suicidal ideation and concurrent depression and suicidal ideation (i.e. women who reported having both depression and suicidal ideation at a given visit) as a dependent variable, overall patterns of association across menopausal stages were similar to the findings of the original analyses. However, given the lack of information on the exact timing of suicidal ideation with the timeframe of the inquiry being within the past 12 months, we cannot rule out the possibility that some women may have reported the events that had occurred in the prior stage. Further large-scale longitudinal studies are warranted to confirm the association between MT and suicidality. Lastly, our study subjects consisted mostly of relatively healthy Korean women with high socioeconomic status, which may limit the generalisability of our findings to other populations with different races/ethnicities or demographic characteristics.

The present study has several strengths. First, this was a large-scale study that examined women's psychological health across menopausal stages in Korea. To our knowledge, this is the first study to examine women's psychological health based on the four distinctive menopausal stages in an Asian population. Second, this study used the STRAW + 10, which is known to be the current standard measure for classifying the specific menopausal stages. Because most women's health studies, especially concerning menopause, are based on dichotomous classification, either menopausal or non-menopausal, our study has the great advantage of identifying the menopausal stage and the potential health complications at a certain stage. Third, a wide range of data was obtained through standardised methods, enabling us to examine independent associations between menopausal stages and depressive symptoms.

In conclusion, in this study of middle-aged women, the prevalence of depressive symptoms and suicidality gradually increased with the advancing menopausal stage. Thus, psychological vulnerability around the time of MT should be considered, and a better understanding of the association between menopausal stages and women's mental health may help identify women at risk for depression and suicidality.

## Data Availability

The data are not available to be shared publicly because we do not have permission from the IRB to distribute the data. However, analytical methods are available from the corresponding author on reasonable request.

## Supplementary material

For supplementary material accompanying this paper visit https://doi.org/10.1017/S2045796022000439. click here to view supplementary material

## References

[ref1] Albert PR (2015) Why is depression more prevalent in women? Journal of Psychiatry & Neuroscience : JPN 40, 219–221. 26107348 10.1503/jpn.150205 PMC4478054

[ref2] Alvaro PK, Roberts RM and Harris JK (2013) A systematic review assessing bidirectionality between sleep disturbances, anxiety, and depression. Sleep 36, 1059–1068. 23814343 10.5665/sleep.2810 PMC3669059

[ref3] Arango V, Underwood MD, Boldrini M, Tamir H, Kassir SA, Hsiung S, Chen JJ and Mann JJ (2001) Serotonin 1A receptors, serotonin transporter binding and serotonin transporter mRNA expression in the brainstem of depressed suicide victims. Neuropsychopharmacology 25, 892–903. 11750182 10.1016/S0893-133X(01)00310-4

[ref4] Baca-Garcia E, Diaz-Sastre C, Ceverino A, Perez-Rodriguez MM, Navarro-Jimenez R, Lopez-Castroman J, Saiz-Ruiz J, Leon JD and Oquendo MA (2010) Suicide attempts among women during low estradiol/low progesterone states. Journal of Psychiatric Research 44, 209–214. 19782376 10.1016/j.jpsychires.2009.08.004

[ref5] Bailey RK, Mokonogho J and Kumar A (2019) Racial and ethnic differences in depression: current perspectives. Neuropsychiatric Disease and Treatment 15, 603–609. 30863081 10.2147/NDT.S128584 PMC6390869

[ref6] Berman NE, Puri V, Chandrala S, Puri S, Macgregor R, Liverman CS and Klein RM (2006) Serotonin in trigeminal ganglia of female rodents: relevance to menstrual migraine. Headache 46, 1230–1245. 16942467 10.1111/j.1526-4610.2006.00528.x

[ref7] Blumel JE, Chedraui P, Aedo S, Fica J, Mezones-Holguin E, Baron G, Bencosme A, Benitez Z, Bravo LM, Calle A, Flores D, Espinoza MT, Gomez G, Hernandez-Bueno JA, Laribezcoa F, Martino M, Lima S, Monterrosa A, Mostajo D, Ojeda E, Onatra W, Sanchez H, Tserotas K, Vallejo MS, Witis S and Zuniga MC (2015) Obesity and its relation to depressive symptoms and sedentary lifestyle in middle-aged women. Maturitas 80, 100–105. 25459364 10.1016/j.maturitas.2014.10.007

[ref8] Boden JM and Fergusson DM (2011) Alcohol and depression. Addiction 106, 906–914. 21382111 10.1111/j.1360-0443.2010.03351.x

[ref9] Bromberger JT and Kravitz HM (2011) Mood and menopause: findings from the Study of Women's Health Across the Nation (SWAN) over 10 years. Obstetrics and Gynecology Clinics of North America 38, 609–625. 21961723 10.1016/j.ogc.2011.05.011 PMC3197240

[ref10] Bromberger JT, Matthews KA, Schott LL, Brockwell S, Avis NE, Kravitz HM, Everson-Rose SA, Gold EB, Sowers M and Randolph Jr JF (2007) Depressive symptoms during the menopausal transition: the Study of Women's Health Across the Nation (SWAN). Journal of Affective Disorders 103, 267–272. 17331589 10.1016/j.jad.2007.01.034 PMC2048765

[ref11] Bromberger JT, Kravitz HM, Chang YF, Cyranowski JM, Brown C and Matthews KA (2011) Major depression during and after the menopausal transition: Study of Women's Health Across the Nation (SWAN). Psychological Medicine 41, 1879–1888. 21306662 10.1017/S003329171100016X PMC3584692

[ref12] Carretti N, Florio P, Bertolin A, Costa CV, Allegri G and Zilli G (2005) Serum fluctuations of total and free tryptophan levels during the menstrual cycle are related to gonadotrophins and reflect brain serotonin utilization. Human Reproduction 20, 1548–1553. 15831515 10.1093/humrep/deh795

[ref13] Chang Y, Ryu S, Choi Y, Zhang Y, Cho J, Kwon MJ, Hyun YY, Lee KB, Kim H, Jung HS, Yun KE, Ahn J, Rampal S, Zhao D, Suh BS, Chung EC, Shin H, Pastor-Barriuso R and Guallar E (2016) Metabolically healthy obesity and development of chronic kidney disease: a cohort study. Annals of Internal Medicine 164, 305–312. 26857595 10.7326/M15-1323

[ref14] Cho MJ and Kim KH (1998) Use of the center for epidemiologic studies depression (CES-D) scale in Korea. Journal of Nervous and Mental Disease 186, 304–310. 9612448 10.1097/00005053-199805000-00007

[ref15] Cohen LS, Soares CN, Vitonis AF, Otto MW and Harlow BL (2006) Risk for new onset of depression during the menopausal transition: the Harvard study of moods and cycles. Archives of General Psychiatry 63, 385–390. 16585467 10.1001/archpsyc.63.4.385

[ref16] Colvin A, Richardson GA, Cyranowski JM, Youk A and Bromberger JT (2017) The role of family history of depression and the menopausal transition in the development of major depression in midlife women: study of women's health across the nation mental health study (SWAN MHS). Depression and Anxiety 34, 826–835. 28489293 10.1002/da.22651 PMC5585035

[ref17] Derby CA, Crawford SL, Pasternak RC, Sowers M, Sternfeld B and Matthews KA (2009) Lipid changes during the menopause transition in relation to age and weight: the Study of Women's Health Across the Nation. American Journal of Epidemiology 169, 1352–1361. 19357323 10.1093/aje/kwp043 PMC2727246

[ref18] Eichling PS and Sahni J (2005) Menopause related sleep disorders. Journal of Clinical Sleep Medicine 1, 291–300.17566192

[ref19] Farmer ME, Locke BZ, Moscicki EK, Dannenberg AL, Larson DB and Radloff LS (1988) Physical activity and depressive symptoms: the NHANES I Epidemiologic Follow-up Study. American Journal of Epidemiology 128, 1340–1351. 3264110 10.1093/oxfordjournals.aje.a115087

[ref20] Flensborg-Madsen T, Mortensen EL, Knop J, Becker U, Sher L and Gronbaek M (2009) Comorbidity and temporal ordering of alcohol use disorders and other psychiatric disorders: results from a Danish register-based study. Comprehensive Psychiatry 50, 307–314. 19486728 10.1016/j.comppsych.2008.09.003

[ref21] Freeman EW, Sammel MD, Lin H and Nelson DB (2006) Associations of hormones and menopausal status with depressed mood in women with no history of depression. Archives of General Psychiatry 63, 375–382. 16585466 10.1001/archpsyc.63.4.375

[ref22] Freeman EW, Sammel MD, Boorman DW and Zhang R (2014) Longitudinal pattern of depressive symptoms around natural menopause. JAMA Psychiatry 71, 36–43. 24227182 10.1001/jamapsychiatry.2013.2819 PMC4576824

[ref23] Friedrich MJ (2017) Depression is the leading cause of disability around the world. JAMA 317, 1517. 10.1001/jama.2017.3826 28418490

[ref24] Harlow SD, Gass M, Hall JE, Lobo R, Maki P, Rebar RW, Sherman S, Sluss PM, de Villiers TJ and Group SC (2012) Executive summary of the Stages of Reproductive Aging Workshop + 10: addressing the unfinished agenda of staging reproductive aging. Journal of Clinical Endocrinology and Metabolism 97, 1159–1168. 22344196 10.1210/jc.2011-3362 PMC3319184

[ref25] Hofmeister S and Bodden S (2016) Premenstrual syndrome and premenstrual dysphoric disorder. American Family Physician 94, 236–240. 27479626

[ref26] Jasienska G, Ziomkiewicz A, Gorkiewicz M and Pajak A (2005) Body mass, depressive symptoms and menopausal status: an examination of the ‘Jolly Fat’ hypothesis. Women's Health Issues 15, 145–151. 15894200 10.1016/j.whi.2005.02.002

[ref27] Kékes Szabó M (2015) The relationship between body image and self-esteem. European Psychiatry 30, 1354.

[ref28] Kim MD, Hong SC, Lee CI, Kwak YS, Shin TK, Jang YH, Oh EH, Lee JW, Jeon BH and Hwang SE (2007) Prevalence of depression and correlates of depressive symptoms for residents in the urban part of Jeju Island, Korea. International Journal of Social Psychiatry 53, 123–134. 17472086 10.1177/0020764006075022

[ref29] Klimes-Dougan B, Safer MA, Ronsaville D, Tinsley R and Harris SJ (2007) The value of forgetting suicidal thoughts and behavior. Suicide and Life-Threatening Behavior 37, 431–438. 17896883 10.1521/suli.2007.37.4.431

[ref30] Koo S, Ahn Y, Lim JY, Cho J and Park HY (2017) Obesity associates with vasomotor symptoms in postmenopause but with physical symptoms in perimenopause: a cross-sectional study. BMC Women's Health 17, 126. 29216853 10.1186/s12905-017-0487-7 PMC5721621

[ref31] Kornstein SG, Young EA, Harvey AT, Wisniewski SR, Barkin JL, Thase ME, Trivedi MH, Nierenberg AA and Rush AJ (2010) The influence of menopause status and postmenopausal use of hormone therapy on presentation of major depression in women. Menopause 17, 828–839. 20616669 10.1097/gme.0b013e3181d770a8 PMC2949279

[ref32] Kozakowski J, Gietka-Czernel M, Leszczynska D and Majos A (2017) Obesity in menopause – our negligence or an unfortunate inevitability? Przeglad Menopauzalny – Menopause Review 16, 61–65. 28721132 10.5114/pm.2017.68594 PMC5509974

[ref33] Kulkarni J (2018) Perimenopausal depression – an under-recognised entity. Australian Prescriber 41, 183–185. 30670885 10.18773/austprescr.2018.060 PMC6299176

[ref34] Luckett T, Butow PN, King MT, Oguchi M, Heading G, Hackl NA, Rankin N and Price MA (2010) A review and recommendations for optimal outcome measures of anxiety, depression and general distress in studies evaluating psychosocial interventions for English-speaking adults with heterogeneous cancer diagnoses. Supportive Care in Cancer 18, 1241–1262. 20596731 10.1007/s00520-010-0932-8

[ref35] Maki PM, Kornstein SG, Joffe H, Bromberger JT, Freeman EW, Athappilly G, Bobo WV, Rubin LH, Koleva HK, Cohen LS, Soares CN and Board of Trustees for The North American Menopause Society and the Women and Mood Disorders Task Force of the National Network of Depression Centers (2018) Guidelines for the evaluation and treatment of perimenopausal depression: summary and recommendations. Menopause 25, 1069–1085. 30179986 10.1097/GME.0000000000001174

[ref36] McQueen JK, Wilson H and Fink G (1997) Estradiol-17 beta increases serotonin transporter (SERT) mRNA levels and the density of SERT-binding sites in female rat brain. Brain Research. Molecular Brain Research 45, 13–23. 9105666 10.1016/s0169-328x(96)00233-1

[ref37] Milic J, Glisic M, Voortman T, Borba LP, Asllanaj E, Rojas LZ, Troup J, Kiefte-de Jong JC, van Beeck E, Muka T and Franco OH (2018) Menopause, ageing, and alcohol use disorders in women. Maturitas 111, 100–109. 29673827 10.1016/j.maturitas.2018.03.006

[ref38] Murphy MM, Verjee MA, Bener A and Gerber LM (2013) The hopeless age? A qualitative exploration of the experience of menopause in Arab women in Qatar. Climacteric 16, 550–554. 23374139 10.3109/13697137.2013.771330

[ref39] Noh JW, Kwon YD, Yang Y, Cheon J and Kim J (2018) Relationship between body image and weight status in east Asian countries: comparison between South Korea and Taiwan. BMC Public Health 18, 814. 29970058 10.1186/s12889-018-5738-5 PMC6029392

[ref40] Norstrom T and Rossow I (2016) Alcohol consumption as a risk factor for suicidal behavior: a systematic review of associations at the individual and at the population level. Archives of Suicide Research 20, 489–506. 26953621 10.1080/13811118.2016.1158678

[ref41] Oh JY, Yang YJ, Kim BS and Kang JH (2007) Validity and reliability of Korean version of International Physical Activity Questionnaire (IPAQ) Short Form. Journal of the Korean Academy of Family Medicine 28, 532–541.

[ref42] Oquendo MA and Mann JJ (2001) Identifying and managing suicide risk in bipolar patients. Journal of Clinical Psychiatry 62(Suppl 25), 31–34. 11765094

[ref43] Peltier MR, Verplaetse TL, Roberts W, Moore K, Burke C, Marotta PL, Phillips S, Smith PH and McKee SA (2020) Changes in excessive alcohol use among older women across the menopausal transition: a longitudinal analysis of the Study of Women's Health Across the Nation. Biology of Sex Differences 11, 37. 32665024 10.1186/s13293-020-00314-7 PMC7362573

[ref44] Pinto-Meza A, Usall J, Serrano-Blanco A, Suarez D and Haro JM (2006) Gender differences in response to antidepressant treatment prescribed in primary care. Does menopause make a difference? Journal of Affective Disorders 93, 53–60. 16549204 10.1016/j.jad.2006.02.010

[ref45] Ryu S, Chang Y, Jung HS, Yun KE, Kwon MJ, Choi Y, Kim CW, Cho J, Suh BS, Cho YK, Chung EC, Shin H and Kim YS (2015) Relationship of sitting time and physical activity with non-alcoholic fatty liver disease. Journal of Hepatology 63, 1229–1237. 26385766 10.1016/j.jhep.2015.07.010

[ref46] Schreiber DR and Dautovich ND (2017) Depressive symptoms and weight in midlife women: the role of stress eating and menopause status. Menopause 24, 1190–1199. 28697039 10.1097/GME.0000000000000897 PMC5607068

[ref47] Smith KF, Huber LR, Issel LM and Warren-Findlow J (2015) The association between maternal depression during pregnancy and adverse birth outcomes: a retrospective cohort study of PRAMS participants. Journal of Community Health 40, 984–992. 25833420 10.1007/s10900-015-0022-4

[ref48] Soares CN and Zitek B (2008) Reproductive hormone sensitivity and risk for depression across the female life cycle: a continuum of vulnerability? Journal of Psychiatry & Neuroscience: JPN 33, 331–343. 18592034 PMC2440795

[ref49] Sullivan LE, Fiellin DA and O'Connor PG (2005) The prevalence and impact of alcohol problems in major depression: a systematic review. The American Journal of Medicine 118, 330–341. 15808128 10.1016/j.amjmed.2005.01.007

[ref50] Timur S and Sahin NH (2010) The prevalence of depression symptoms and influencing factors among perimenopausal and postmenopausal women. Menopause 17, 545–551. 20400922 10.1097/gme.0b013e3181cf8997

[ref51] Tseng LA, El Khoudary SR, Young EA, Farhat GN, Sowers M, Sutton-Tyrrell K and Newman AB (2012) The association of menopause status with physical function: the Study of Women's Health Across the Nation. Menopause 19, 1186–1192. 22760087 10.1097/gme.0b013e3182565740 PMC3526111

[ref52] Voss C, Ollmann TM, Miché M, Venz J, Hoyer J, Pieper L, Höfler M and Beesdo-Baum K (2019) Prevalence, onset, and course of suicidal behavior among adolescents and young adults in Germany. JAMA Network Open 2, e1914386. 31664450 10.1001/jamanetworkopen.2019.14386 PMC6824228

[ref53] Woods NF, Smith-DiJulio K, Percival DB, Tao EY, Mariella A and Mitchell S (2008) Depressed mood during the menopausal transition and early postmenopause: observations from the Seattle Midlife Women's Health Study. Menopause 15, 223–232. 18176355 10.1097/gme.0b013e3181450fc2

[ref54] Worsley R, Bell R, Kulkarni J and Davis SR (2014) The association between vasomotor symptoms and depression during perimenopause: a systematic review. Maturitas 77, 111–117. 24365649 10.1016/j.maturitas.2013.11.007

[ref55] Yi S-W, Jung M, Kimm H, Sull J-W, Lee E, Lee KO and Ohrr H (2016) Usual alcohol consumption and suicide mortality among the Korean elderly in rural communities: Kangwha Cohort Study. Journal of Epidemiology and Community Health 70, 778–783. 26888918 10.1136/jech-2015-206849 PMC4975804

