# Supplementary material for: Depressive symptoms and suicidality by menopausal stages among middle-aged Korean women
Source: Epidemiol Psychiatr Sci. 2022 Aug 26;31:e60. doi: 10.1017/S2045796022000439 (PMC9428901; doi:10.1017/S2045796022000439)
Supplement: Supplementary file 1 [file S2045796022000439sup001.docx]

**Table S1. Baseline characteristics of study participants by menopausal stage**

| **Characteristics** | **Overall** | **Menopausal stages** | | | | ***p* for trend** |
| --- | --- | --- | --- | --- | --- | --- |
|  |  | **Pre-menopause** | **Early transition** | **Late transition** | **Post-menopause** |  |
| Number of participants | 45,177 | 22,454 | 7,001 | 3,924 | 11,788 |  |
| Age (years)^e^ | 47.3 (6.5) | 43.5 (2.7) | 44.3 (3.1) | 47.1 (3.8) | 56.2 (4.9) | <0.001 |
| Early menarche (%) ^a^ | 3.0 | 4.0 | 3.4 | 2.6 | 0.9 | <0.001 |
| Parity (%)^b^ | 92.7 | 91.7 | 90.4 | 91.3 | 96.7 | <0.001 |
| Current smoker (%) | 1.8 | 1.8 | 2.1 | 1.7 | 1.7 | 0.425 |
| Alcohol intake (%)^c^ | 10.8 | 11.4 | 12.2 | 10.5 | 8.9 | <0.001 |
| HEPA (%) | 15.2 | 13.3 | 13.6 | 13.8 | 20.1 | <0.001 |
| Education level (%)^d^ | 70.3 | 80.6 | 80.4 | 74.8 | 42.6 | <0.001 |
| Diabetes (%) | 4.1 | 1.9 | 2.1 | 3.4 | 9.6 | <0.001 |
| Hypertension (%) | 9.6 | 4.5 | 5.3 | 9.5 | 22.0 | <0.001 |
| Dyslipidemia medication (%) | 4.7 | 1.1 | 1.6 | 2.9 | 14.1 | <0.001 |
| Poor sleep quality (%) | 19.2 | 16.1 | 21.5 | 21.2 | 23.2 | <0.001 |
| Sleep duration (h/day) | 6.56 (1.13) | 6.63 (1.07) | 6.53 (1.11) | 6.40 (1.12) | 6.49 (1.23) | <0.001 |
| Body mass index (kg/m^2^) ^e^ | 22.6 (3.1) | 22.2 (3.0) | 22.2 (3.1) | 23.0 (3.4) | 23.3 (3.1) | <0.001 |
| Systolic BP (mmHg) ^e^ | 106.7 (12.7) | 104.3 (11.3) | 104.8 (11.7) | 107.8 (13.6) | 112.0 (13.8) | <0.001 |
| Diastolic BP (mmHg) ^e^ | 67.8 (9.2) | 66.5 (8.8) | 66.8 (9.0) | 68.7 (9.8) | 70.6 (9.3) | <0.001 |
| Glucose (mg/dl) ^e^ | 94.5 (14.2) | 92.8 (11.9) | 93.0 (12.1) | 94.3 (14.7) | 98.8 (17.8) | <0.001 |
| Total cholesterol (mg/dl)^e^ | 195.7 (34.0) | 189.8 (30.8) | 191.3 (30.9) | 201.5 (34.5) | 207.7 (37.7) | <0.001 |
| LDL-C (mg/dl) ^e^ | 124.9 (32.8) | 118.5 (29.1) | 119.7 (29.3) | 129.8 (33.1) | 138.7 (36.6) | <0.001 |
| HDL-C (mg/dl) ^e^ | 66.3 (16.3) | 67.1 (15.9) | 67.3 (16.3) | 66.5 (16.3) | 64.1 (16.8) | <0.001 |
| Triglycerides (mg/dl)^f^ | 79 (59–109) | 75 (57–100) | 75 (57–102) | 83 (62–118) | 90 (65–125) | <0.001 |
| ALT (u/l) ^f^ | 15 (12–20) | 13 (11–17) | 14 (11–18) | 15 (12–21) | 19 (15–26) | <0.001 |
| GGT (u/l) ^f^ | 14 (11–20) | 13 (11–18) | 13 (11–19) | 15 (11–21) | 18 (13–26) | <0.001 |
| hsCRP (mg/L) ^f^ | 0.04 (0.02–0.07) | 0.03 (0.02–0.06) | 0.04 (0.02–0.07) | 0.04 (0.03–0.09) | 0.05 (0.03–0.09) | <0.001 |
| HOMA-IR^f^ | 1.22 (0.81–1.82) | 1.21 (0.81–1.78) | 1.17 (0.78–1.74) | 1.17 (0.79–1.80) | 1.28 (0.83–1.98) | <0.001 |
| Total energy intake (kcal/d)^f, g^ | 1119.1 (804.6–1481.3) | 1089.6 (784–1444.5) | 1100.8 (793.2–1462.2) | 1079.7 (785.1–1447.7) | 1228.6 (891.3–1592.3) | <0.001 |

^a^<12 years; ^b^≥3 times; ^c^≥10 g of ethanol per day; ^d^≥college graduate; data are expressed as ^e^means (standard deviations), ^f^median (interquartile ranges), or percentages ^g^among 45,177 participants with plausible estimated energy intake levels (within three standard deviations from the log-transformed mean energy intake).

Abbreviations: ALT, alanine aminotransferase; BP, blood pressure; HDL-C, high-density lipoprotein-cholesterol; HEPA, health-enhancing physical activity; HOMA-IR, homeostasis model assessment of insulin resistance; LDL-C, low-density lipoprotein cholesterol.

**Table S2.** **Prevalence ratios^a^ (95% CI) of depression by menopausal stage after further adjustment for the history of suicidal behavior**

|  | Menopausal stages | | | | *p* for trend |
| --- | --- | --- | --- | --- | --- |
|  | Pre-menopause | Early transition | Late transition | Post-menopause |  |
| Number | 22,454 | 7,011 | 3,924 | 11,788 |  |
| CESD score 16-24 |  |  |  |  |  |
| Multivariate-adjusted PR^a^ | 1.00 (reference) | 1.28 (1.16-1.42) | 1.21 (1.05-1.38) | 1.58 (1.36-1.84) | <0.001 |
| CESD score ≥25 |  |  |  |  |  |
| Multivariate-adjusted PR^a^ | 1.00 (reference) | 1.31 (1.11-1.55) | 1.39 (1.13-1.73) | 1.86 (1.46-2.37) | <0.001 |

^a^Estimated from multinomial logistic regression models using CESD scores as outcomes categorized as <16, 16 – 24, and ≥25. Multivariable Model 1 was adjusted for age, center, year of screening examination, smoking status, alcohol intake, physical activity level, total energy intake, body mass index, educational level, antihypertensive medication, parity, age at menarche, sleep duration, sleep quality and history of suicidal behavior

Abbreviation: CESD, Center for Epidemiological Studies-Depression; CI, confidence interval; PR, prevalence ratio

**Table S3. Odds ratios^a^ (95% CI) of suicidal ideation by menopausal stage after further adjustment for history of suicidal behavior**

|  | Menopausal stages | | | | *p* for trend |
| --- | --- | --- | --- | --- | --- |
|  | Pre-menopause | Early transition | Late transition | Post-menopause |  |
| Number | 22,454 | 7,011 | 3,924 | 11,788 |  |
| Multivariate-adjusted OR^a^ | 1.00 (reference) | 1.25 (1.12-1.39) | 1.07 (0.93-1.24) | 1.45 (1.24-1.69) | <0.001 |

^a^Estimated from binomial logistic regression models. Multivariable Model was adjusted for age, center, year of a screening exam, smoking status, alcohol intake, physical activity level, total energy intake, body mass index, educational level, parity, age at menarche, sleep duration, sleep quality, and history of suicidal behavior.

Abbreviation: CI, confidence interval; OR, odds ratio

**Table S4.** **Odds ratios^a^ (95% CI) of suicidal behavior by depression and suicidal ideation according to menopausal stage**

|  | Multivariate-adjusted OR^a^ (95% CI) of suicidal behavior | | | | | | | |
| --- | --- | --- | --- | --- | --- | --- | --- | --- |
|  | Cases (%) | Pre-menopause  (N=22,454) | Cases (%) | Early transition  (N=7,011) | Cases (%) | Late transition (N=3,924) | Cases (%) | Post-menopause (N=11,788) |
| CESD score |  |  |  |  |  |  |  |  |
| <16 | 9 (0.04) | 1.00 (reference) | 2 (0.03) | 1.00 (reference) | 2 (0.06) | 1.00 (reference) | 41 (0.40) | 1.00 (reference) |
| 16-24 | 4 (0.29) | 3.01 (0.77-11.79) | 0 (0) | - | 0 (0) | - | 7 (0.60) | 1.37 (0.59-3.16) |
| ≥25 | 7 (1.46) | 15.55 (5.47-50.07) | 3 (1.39) | 67.14 (4.53-994.52) | 2 (1.63) | - | 7 (1.54) | 3.45 (1.42-8.36) |
| *p* for trend |  | <0.001 |  | 0.003 |  | - |  | 0.011 |
| Suicidal ideation |  |  |  |  |  |  |  |  |
| No | 0 (0) | 1.00 (reference) | 0 (0) | 1.00 (reference) | 0 (0) | 1.00 (reference) | 9 (0.08) | 1.00 (reference) |
| Yes | 20 (1.56) | - | 5 (0.94) | - | 4 (0.10) | - | 46 (3.97) | 47.55 (22.53-100.34) |

^a^ Estimated from binomial logistic regression models. Multivariable Model was adjusted for age, center, year of a screening exam, smoking status, alcohol intake, physical activity level, total energy intake, body mass index, educational level, parity, age at menarche, sleep duration, and quality

Abbreviation: CESD, Center for Epidemiological Studies-Depression; CI, confidence interval; PR, prevalence ratio

**Table S5.** **Associations between menopausal stages, depression, and suicidal ideation among participants without excluding subjects with a history of mental illness diagnosis (n=2,357) or currently taking anxiety drugs (n=703), antidepressants, or other neuropsychological medications (n=869)**

|  | Menopausal stages | | | | *p* for trend |
| --- | --- | --- | --- | --- | --- |
|  | Pre-menopause  n = 23,238 | Early transition  n = 7349 | Late transition  n = 4118 | Post-menopause  n = 12519 |  |
| **Depressive symptoms** |  |  |  |  |  |
| CESD score 16-24 |  |  |  |  |  |
| Multivariate-adjusted PR^a^ |  |  |  |  |  |
| **Without** adjustment for psychiatric history^b^ | 1.00 (reference) | 1.27 (1.15-1.41) | 1.21 (1.06-1.38) | 1.59 (1.38-1.84) | <0.001 |
| **With** adjustment for psychiatric history^c^ | 1.00 (reference) | 1.27 (1.15-1.40) | 1.21 (1.06-1.37) | 1.59 (1.38-1.84) | <0.001 |
| CESD score ≥25 |  |  |  |  |  |
| Multivariate-adjusted PR^a^ |  |  |  |  |  |
| **Without** adjustment for psychiatric history^b^ | 1.00 (reference) | 1.26 (1.08-1.47) | 1.35 (1.12-1.64) | 1.67 (1.34-2.08) | <0.001 |
| **With** adjustment for psychiatric history^c^ | 1.00 (reference) | 1.26 (1.08-1.46) | 1.34 (1.10-1.62) | 1.69 (1.36-2.11) | <0.001 |
| **Suicidal ideation** |  |  |  |  |  |
| Multivariate-adjusted OR^d^ |  |  |  |  |  |
| **Without** adjustment for psychiatric history^b^ | 1.00 (reference) | 1.26 (1.14-1.39) | 1.07 (0.93-1.22) | 1.43 (1.23-1.65) | <0.001 |
| **With** adjustment for psychiatric history^c^ | 1.00 (reference) | 1.26 (1.14-1.39) | 1.06 (0.93-1.22) | 1.43 (1.24-1.66) | <0.001 |

^a^Estimated from multinomial logistic regression models using CESD scores as outcomes categorized as <16, 16 – 24, and ≥25. Multivariable Model 1 was adjusted for age, center, year of screening examination, smoking status, alcohol intake, physical activity level, total energy intake, body mass index, educational level, antihypertensive medication, parity, age at menarche, sleep duration, and quality; ^b^ plus a further adjustment for a history of mental illness, anxiety drugs, and neuropsychological medications

^d^Estimated from binomial logistic regression models.

Abbreviation: CESD, Center for Epidemiological Studies-Depression; CI, confidence interval; OR, odds ratio; PR, prevalence ratio

**Table S6.** **Associations between menopausal stages, depression, and suicidal ideation comparing the early transition stage to postmenopausal stage with premenopausal stage**

|  | Menopausal stages | | *p* value |
| --- | --- | --- | --- |
|  | Pre-menopause  n = 22,454 | Early transition to Post-menopause  n = 22,723 |  |
| **Depressive symptoms** |  |  |  |
| CESD score 16-24 |  |  |  |
| Multivariate-adjusted PR (95% CI)^a^ | 1.00 (reference) | 1.30 (1.19-1.42) | <0.001 |
| CESD score ≥25 |  |  |  |
| Multivariate-adjusted PR^a^ | 1.00 (reference) | 1.40 (1.22-1.61) | <0.001 |
| **Suicidal ideation** |  |  |  |
| Multivariate-adjusted OR^b^ | 1.00 (reference) | 1.23 (1.12-1.35) | <0.001 |

^a^Estimated from multinomial logistic regression models using CESD scores as outcomes categorized as <16, 16 – 24, and ≥25.

^b^Estimated from binomial logistic regression models.

Multivariable Model was adjusted for age, center, year of screening examination, smoking status, alcohol intake, physical activity level, total energy intake, body mass index, educational level, antihypertensive medication, parity, age at menarche, sleep duration, and quality

Abbreviation: CESD, Center for Epidemiological Studies-Depression; CI, confidence interval; OR, odds ratio; PR, prevalence ratio

**Table S7.** **Associations between menopausal stages, depression, and suicidal ideation comparing post-menopasual stage to the earlier stages (from premenopausal stage to late transition stage)**

|  | Menopausal stages | | *p*-value |
| --- | --- | --- | --- |
|  | Pre-menopause to late transition  n = 33,389 | Post-menopause  n = 11,788 |  |
| **Depressive symptoms** |  |  |  |
| CESD score 16-24 |  |  |  |
| Multivariate-adjusted PR (95% CI)^a^ | 1.00 (reference) | 1.39 (1.21-1.60) | <0.001 |
| CESD score ≥25 |  |  |  |
| Multivariate-adjusted PR^a^ | 1.00 (reference) | 1.56 (1.25-1.95) | <0.001 |
| **Suicidal ideation** |  |  |  |
| Multivariate-adjusted OR^b^ | 1.00 (reference) | 1.35 (1.17-1.56) | <0.001 |

^a^Estimated from multinomial logistic regression models using CESD scores as outcomes categorized as <16, 16 – 24, and ≥25.

^b^Estimated from binomial logistic regression models.

Multivariable Model was adjusted for age, center, year of screening examination, smoking status, alcohol intake, physical activity level, total energy intake, body mass index, educational level, antihypertensive medication, parity, age at menarche, sleep duration, and quality

Abbreviation: CESD, Center for Epidemiological Studies-Depression; CI, confidence interval; OR, odds ratio; PR, prevalence ratio

**Table S8.** **Prevalence ratios^a^ (95% CI) of depression and suicidal ideation by menopausal stage**

|  | Menopausal stages | | | | *p* for trend |
| --- | --- | --- | --- | --- | --- |
|  | Pre-menopause | Early transition | Late transition | Post-menopause |  |
| Number | 22,454 | 7,011 | 3,924 | 11,788 |  |
| Isolated depression (CESD score ≥16) |  |  |  |  |  |
| Multivariate-adjusted PR^a^ | 1.00 (reference) | 1.25 (1.13-1.40) | 1.28 (1.11-1.46) | 1.61 (1.38-1.88) | <0.001 |
| Isolated suicidal ideation |  |  |  |  |  |
| Multivariate-adjusted PR^a^ | 1.00 (reference) | 1.17 (1.02-1.35) | 1.02 (0.84-1.24) | 1.33 (1.08-1.64) | 0.014 |
| Both depression (CESD score ≥16) and suicidal ideation |  |  |  |  |  |
| Multivariate-adjusted PR^a^ | 1.00 (reference) | 1.41 (1.21-1.64) | 1.20 (0.98-1.48) | 1.82 (1.46-2.27) | <0.001 |

^a^Estimated from multinomial logistic regression models using CESD scores as outcomes categorized as <16, 16 – 24, and ≥25. Multivariable Model 1 was adjusted for age, center, year of screening examination, smoking status, alcohol intake, physical activity level, total energy intake, body mass index, educational level, antihypertensive medication, parity, age at menarche, sleep duration, and quality

Abbreviation: CESD, Center for Epidemiological Studies-Depression; CI, confidence interval; PR, prevalence ratio

**Table S9.** **Odds ratios^a^ (95% CI) of depression (CESD score ≥25**) **by menopausal stage in clinically relevant subgroups**

| **Subgroup** | **Menopausal stages** | | | | ***p* for trend** | ***p* for interaction** |
| --- | --- | --- | --- | --- | --- | --- |
|  | **Pre-menopause** | **Early transition** | **Late transition** | **Post-menopause** |  |  |
| **BMI** |  |  |  |  |  | 0.002 |
| < 25 kg/m^2^ (N=36,631) | 1.00 (reference) | 1.26 (1.05–1.52) | 1.36 (1.07–1.74) | 1.99 (1.57–2.53) | <0.001 |  |
| ≥25 kg/m^2^ (N=8,506) | 1.00 (reference) | 2.70 (1.87–3.89) | 2.42 (1.60–3.65) | 2.81 (1.96–4.02) | <0.001 |  |
| **Early menarche** |  |  |  |  |  | 0.617 |
| No (N=43,677) | 1.00 (reference) | 1.44 (1.22–1.71) | 1.57 (1.27–1.94) | 2.12 (1.68–2.67) | <0.001 |  |
| Yes (N=1,333) | 1.00 (reference) | 1.91 (0.85–4.32) | 0.99 (0.23–4.33) | 1.15 (0.26–5.08) | 0.587 |  |
| **Parity** |  |  |  |  |  | 0.124 |
| 0 times (N=3,130) | 1.00 (reference) | 1.44 (0.89–2.34) | 2.47 (1.46–4.18) | 1.54 (0.81–2.92) | 0.008 |  |
| ≥1 time (N=39,944) | 1.00 (reference) | 1.49 (1.24–1.77) | 1.48 (1.17–1.86) | 2.22 (1.74–2.81) | <0.001 |  |
| **Sleep quality** |  |  |  |  |  | 0.069 |
| Good (N=36,368) | 1.00 (reference) | 1.15 (0.88–1.50) | 1.33 (0.96–1.83) | 2.01 (1.53–2.64) | <0.001 |  |
| Poor (N=8,653) | 1.00 (reference) | 1.38 (1.11–1.72) | 1.41 (1.07–1.85) | 1.57 (1.20–2.06) | 0.001 |  |
| **Smoking** |  |  |  |  |  | 0.129 |
| Non-smoker (N=40,269) | 1.00 (reference) | 1.44 (1.20–1.71) | 1.64 (1.32–2.04) | 2.03 (1.60–2.58) | <0.001 |  |
| Ex- or current smoker (N=3,720) | 1.00 (reference) | 1.64 (1.05–2.56) | 0.86 (0.41–1.83) | 2.73 (1.72–4.34) | <0.001 |  |
| **Alcohol intake** |  |  |  |  |  | 0.743 |
| < 10 g /day (N=37,888) | 1.00 (reference) | 1.38 (1.15–1.66) | 1.54 (1.23–1.94) | 1.91 (1.50–2.44) | <0.001 |  |
| ≥ 10 g/day (N=4,594) | 1.00 (reference) | 1.64 (1.09–2.46) | 1.55 (0.90–2.68) | 2.32 (1.55–3.49) | <0.001 |  |
| **HEPA** |  |  |  |  |  | 0.461 |
| No (N=38,256) | 1.00 (reference) | 1.45 (1.22–1.73) | 1.63 (1.31–2.02) | 2.13 (1.68–2.69) | <0.001 |  |
| Yes (N=6,832) | 1.00 (reference) | 1.50 (0.92–2.45) | 0.91 (0.43–1.94) | 1.84 (1.21–2.79) | 0.005 |  |

^a^Estimated from the logistic regression models. Multivariable Model 1 was adjusted for age, center, year of screening examination, smoking status, alcohol intake, physical activity level, total energy intake, BMI, educational level, parity, and age at menarche.

Abbreviation: BMI, body mass index; CESD, Center for Epidemiological Studies-Depression; HEPA, health-enhancing physical activity

**Table S10.** **Odds ratios^a^ (95% CI) of suicidal ideation** **by menopausal stage in clinically relevant subgroups**

| **Subgroup** | **Menopausal stages** | | | | ***p* for trend** | ***p* for interaction** |
| --- | --- | --- | --- | --- | --- | --- |
|  | **Pre-menopause** | **Early transition** | **Late transition** | **Post-menopause** |  |  |
| **BMI** |  |  |  |  |  | 0.285 |
| < 25 kg/m^2^ (N=36,631) | 1.00 (reference) | 1.31 (1.17–1.48) | 1.14 (0.97–1.34) | 1.54 (1.32–1.80) | <0.001 |  |
| ≥25 kg/m^2^ (N=8,506) | 1.00 (reference) | 1.43 (1.12–1.85) | 1.20 (0.90–1.61) | 1.88 (1.51–2.34) | <0.001 |  |
| **Early menarche** |  |  |  |  |  | 0.441 |
| No (N=43,677) | 1.00 (reference) | 1.59 (0.98–2.57) | 0.62 (0.24–1.59) | 1.83 (0.96–3.50) | 0.172 |  |
| Yes (N=1,333) | 1.00 (reference) | 1.32 (1.19–1.47) | 1.17 (1.01–1.35) | 1.61 (1.38–1.87) | <0.001 |  |
| **Parity** |  |  |  |  |  | 0.323 |
| 0 times (N=3,130) | 1.00 (reference) | 1.44 (1.04–1.97) | 1.24 (0.81–1.91) | 1.18 (0.77–1.81) | 0.222 |  |
| ≥1 times (N=39,944) | 1.00 (reference) | 1.35 (1.20–1.51) | 1.17 (1.00–1.36) | 1.68 (1.44–1.97) | <0.001 |  |
| **Sleep quality** |  |  |  |  |  | 0.263 |
| Good (N=36,368) | 1.00 (reference) | 1.32 (1.16–1.51) | 1.08 (0.90–1.30) | 1.55 (1.32–1.83) | <0.001 |  |
| Poor (N=8,653) | 1.00 (reference) | 1.14 (0.95–1.36) | 1.06 (0.85–1.34) | 1.32 (1.09–1.60) | 0.009 |  |
| **Smoking** |  |  |  |  |  | 0.413 |
| Non-smoker (N=40,269) | 1.00 (reference) | 1.36 (1.22–1.52) | 1.13 (0.97–1.31) | 1.55 (1.33–1.82) | <0.001 |  |
| Ex- or current smoker (N=3,720) | 1.00 (reference) | 1.10 (0.81–1.51) | 1.19 (0.79–1.79) | 1.72 (1.26–2.37) | 0.001 |  |
| **Alcohol intake** |  |  |  |  |  | 0.014 |
| < 10 g /day (N=37,888) | 1.00 (reference) | 1.36 (1.21–1.53) | 1.07 (0.91–1.26) | 1.46 (1.24–1.72) | <0.001 |  |
| ≥ 10 g/day (N=4,594) | 1.00 (reference) | 1.21 (0.92–1.59) | 1.45 (1.03–2.04) | 2.00 (1.55–2.60) | <0.001 |  |
| **HEPA** |  |  |  |  |  | 0.222 |
| No (N=38,256) | 1.00 (reference) | 1.35 (1.21–1.51) | 1.11 (0.95–1.29) | 1.57 (1.34–1.83) | <0.001 |  |
| Yes (N=6,832) | 1.00 (reference) | 1.21 (0.88–1.65) | 1.46 (1.01–2.11) | 1.81 (1.41–2.32) | <0.001 |  |

^a^Estimated from the logistic regression models. Multivariable Model 1 was adjusted for age, center, year of screening examination, smoking status, alcohol intake, physical activity level, total energy intake, BMI, educational level, parity, and age at menarche.

Abbreviation: BMI, body mass index; CESD, Center for Epidemiological Studies-Depression; HEPA, health-enhancing physical activity
